# Nasal Exposure to Microcystin Can Trigger Immune Responses in the Lung and Gut Microbiome Dysbiosis via the Lung–Gut Axis: A Pilot Acute Mouse Study

**DOI:** 10.3390/toxins18080333

**Published:** 2026-08-01

**Authors:** Minseung Kim, Sangwoon Chung, John W. Christman, Jiyoung Lee

**Affiliations:** 1 Environmental Sciences Graduate Program, The Ohio State University, Columbus, OH 43210, USA; kim.8548@osu.edu; 2 Department of Internal Medicine, The Ohio State University, Columbus, OH 43210, USA; 3 Division of Environmental Health Sciences, College of Public Health, The Ohio State University, Columbus, OH 43210, USA; 4 Department of Food Science & Technology, The Ohio State University, Columbus, OH 43210, USA; 5 Infectious Diseases Institute, The Ohio State University, Columbus, OH 43210, USA

**Keywords:** harmful algal blooms, cyanotoxin, respiratory exposure, gut microbiome, inflammation, lung–gut axis

## Abstract

Cyanotoxin events are among the most serious consequences of cyanobacterial harmful algal blooms. Microcystins (MCs), among the most prevalent cyanotoxins, are known to adversely affect human health. Recently, respiratory exposure has emerged as an important exposure route for MCs, with potential downstream effects on the gut microbiome through the lung–gut axis. In this pilot study, we investigated acute respiratory immune responses and gut microbiome alterations following MC inhalation using female C57BL/6J mice. Mice were assigned to three dose groups: control (0 µg/kg body weight), medium (25 µg/kg), and high (50 µg/kg). MC-LR was administered intranasally once daily for 3 days. Fecal samples were collected daily for 16S rRNA sequencing, and bronchoalveolar lavage (BAL) fluids were collected following sacrifice for immune cell analysis. MC exposure resulted in significantly increased monocyte counts (*p* < 0.1), while neutrophil, macrophage, and total cell counts did not significantly change (*p* > 0.1), suggesting selective lower respiratory tract inflammation. Functional prediction analysis of gut microbiota revealed significant increases (*p* < 0.1) in pathways associated with host health, including heme biosynthesis, sulfur oxidation, carbon metabolism, and antibiotic resistance. These findings suggest that inhaled MCs may induce respiratory inflammation and contribute to gut microbiome dysbiosis via the lung–gut axis.

## 1. Introduction

Excessive growth of cyanobacteria in waterbodies, known as cyanobacterial harmful algal blooms (CyanoHABs), negatively affects human society in multiple ways. CyanoHABs degrade drinking water quality by releasing odorous compounds [1], increase the burden on water treatment systems, and damage local economies by disrupting fisheries and tourism through the deterioration of aquatic ecosystems [2]. However, the most concerning consequences of CyanoHABs are the production of cyanotoxins, such as microcystins, anatoxin, saxitoxin, cylindrospermopsin, and nodularins [3].

Among these toxins, microcystins (MCs)—cyclic peptides produced by cyanobacterial genera including *Microcystis*, *Planktothrix*, *Dolichospermum*, and *Nostoc*—are the most prevalent and persistent cyanotoxins found in freshwater environments [4]. MC exposure has been associated with hepatotoxicity, neurotoxicity, and dysfunction of the intestinal and reproductive systems in mammals [5,6,7,8]. Mechanistically, MCs inhibit serine/threonine protein phosphatases 1 and 2A and induce oxidative stress, thereby disrupting multiple signaling pathways and contributing to DNA damage, inflammation, and tumor promotion [8]. MC-induced oxidative stress can also trigger apoptosis through both intrinsic mitochondria-mediated pathways [9] and extrinsic death receptor-mediated signaling [10], as well as autophagy [11].

To date, most studies on MC exposure have focused on ingestion through contaminated drinking water and food, as well as dermal contact [12,13]. More recently, inhalation exposures to aerosolized or PM2.5-associated MCs have emerged as an additional concern, with evidence of toxic effects in both human cell lines and animal models. Previous studies have shown that inhaled MC can induce lung injury [14], airway inflammation in mice [15], and mitochondria-mediated apoptosis in human bronchial epithelial cells [16]. Furthermore, Jung et al. [17] demonstrated that inhaled MC can translocate across the respiratory epithelium and affect distal organs, as evidenced by liver congestion in a mouse model.

Inhalation may represent a critical route of MC exposure because humans inhale more than a thousand liters of air daily, which far exceeds the volume of water typically consumed [18]. Once inhaled, the human respiratory system provides a large surface area for toxin absorption [19]. Certain toxins, such as Staphylococcal enterotoxin B, are also known to exhibit greater toxicity through inhalation compared with other exposure routes [20]. Ongoing climate change and accelerating eutrophication are expected to increase the frequency and severity of toxic cyanobacterial bloom events worldwide [21]. Under these conditions, inhalation exposure to MC, together with ingestion and dermal contact, may pose a significant public health concern, given that airborne cyanobacterial derivatives from bloom-affected waters can travel distances of up to 25 km [22].

Despite the growing concern regarding MC exposure via inhalation, substantial knowledge gaps remain. In particular, the toxicological effects of acute, sublethal MC inhalation have not yet been fully characterized. Although previous studies have investigated the lethal dose of inhaled MC and the toxic effects of repeated or prolonged exposure, the biological effects of short-term sublethal inhalation remain largely unexplored. For example, the median lethal dose of MC administered via intranasal instillation was reported to be approximately 250 µg/kg body weight [23]. Similarly, mice receiving a single intranasal dose of 150 µg/kg body weight died within 8 days, whereas administration of 300 µg/kg body weight resulted in mortality within 2 days. In addition to these acute lethality studies, repeated exposure experiments have evaluated the effects of prolonged MC inhalation. Daily intranasal administration of 31 µg/kg body weight for 7 consecutive days induced lung injury in mice [24], while repeated administration of 6.7 µg/kg body weight for 30 days increased polymorphonuclear cell infiltration in the lungs [25]. However, studies specifically investigating the biological effects of short-term sublethal MC inhalation over a limited exposure period have not yet been reported. Addressing this knowledge gap is important because short-term inhalation exposure may occur during recreational activities [26] and occupational activities near bloom-affected waterbodies, such as lifeguarding and fishing [27], potentially contributing to human health risks.

Furthermore, previous studies investigating other exposure pathways, particularly oral exposure through contaminated water, have shown that MC ingestion can alter the gut microbiome and subsequently influence host physiology. For example, in our previous study using a hepatocellular carcinoma mouse model, ingestion of MC-LR or *Microcystis* lysate altered gut microbial structure and reduced microbial alpha diversity [28]. More et al. [29] reported that MC-LR ingestion decreased the abundance of short-chain fatty-acid-producing bacteria in juvenile mice, resulting in the disruption of gut homeostasis and gut barrier integrity. Collectively, these findings indicate that inhaled MC exposure can perturb the gut microbiome through the oral route. It is, therefore, plausible that inhaled MCs may also influence the gut microbiota, potentially through the lung–gut axis. Accumulated evidence supports a bidirectional relationship between the respiratory system and gut microbial communities. For example, inhalation exposure to toxic substances such as particulate matter [30], toluene diisocyanate [31], or co-exposure to carbon nanotube particles and cigarette smoke extract [32] has been shown to alter gut microbiota composition via the lung–gut axis. Moreover, patients with respiratory diseases, including asthma or chronic obstructive pulmonary disease, exhibit gut microbial profiles distinct from those of healthy individuals [33,34,35]. Gut dysbiosis has been implicated in the development of numerous diseases and chronic conditions, including gastrointestinal diseases [36], autoimmune diseases [37], cancers [38], cardiometabolic disorders [39], and neurological disorders [40]. Therefore, inhalation-induced gut dysbiosis may contribute to systemic health effects that extend beyond the respiratory system.

Despite growing concern regarding airborne MC exposure, the effects of nasal MC exposure on the respiratory system and its potential role in inducing gut dysbiosis remain poorly understood. Therefore, in this pilot acute mouse study, we aimed to investigate the immediate respiratory system immune responses and distal gut microbiome alterations following nasal exposure to MC, with particular emphasis on the lung–gut axis.

## 2. Results

### 2.1. Cell Count of Immunocytes

**Figure 1:**
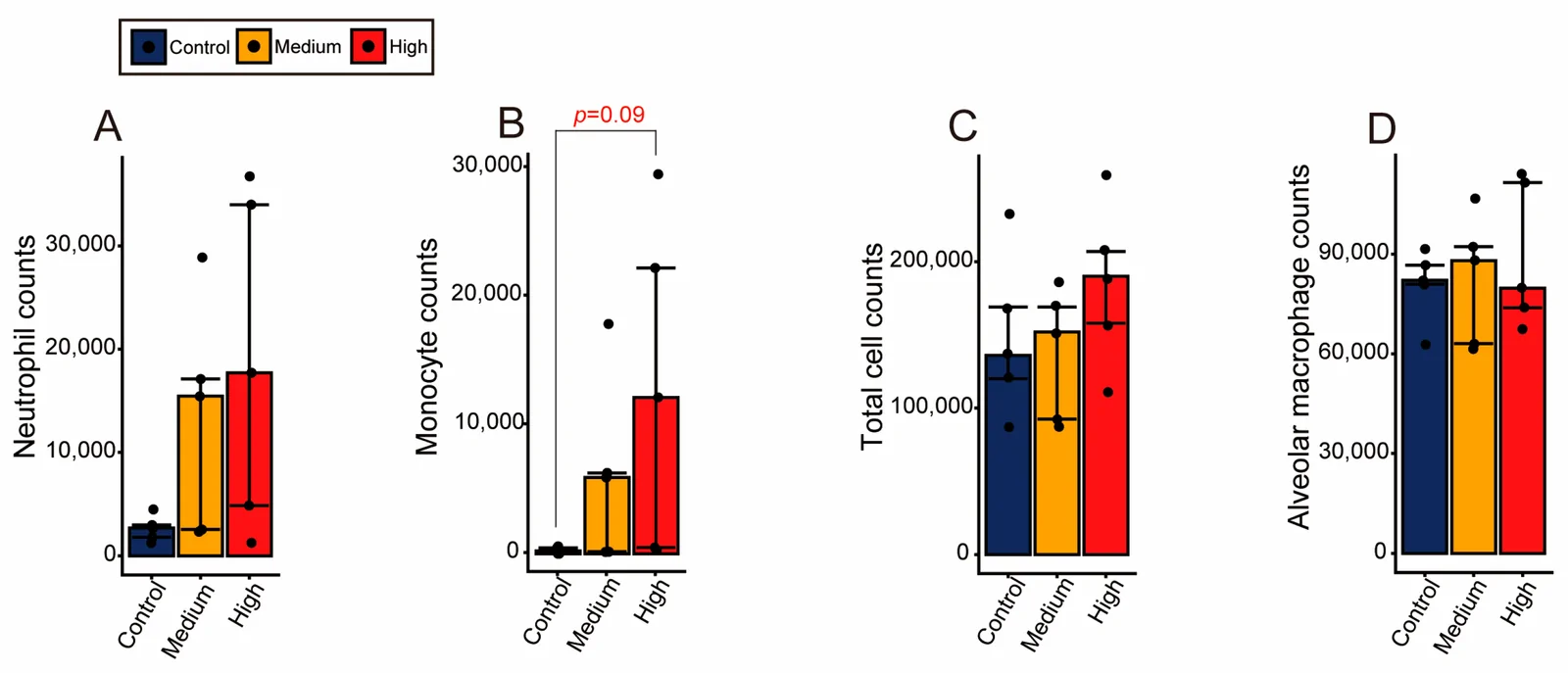
Cell count of immunocytes (*n* = 5 per group). The medians of the counts of neutrophils (**A**), monocytes (**B**), total cells (**C**), and alveolar macrophages (**D**) in bronchoalveolar lavage (BAL) fluid are presented through the bar plots. Jitters represent the value of each measurement, and the error bars indicate the interquartile range.

The number of total cells and immune cells in BAL fluid were counted to determine the immune response in the lower respiratory tract following MC inhalation. The numbers of monocytes in the BAL fluid of the high-dose group significantly increased compared to the control group (Kruskal–Wallis, *p* < 0.1; Dunn test, *p* < 0.1) (Figure 1A and Table A1). The numbers of neutrophils were also observed to follow the increasing trends by MC dose (Figure 1B and Table A2). However, we did not observe significant changes in neutrophils, macrophage, and total cell counts in BAL fluid (Kruskal–Wallis, *p* > 0.1) (Figure 1C,D, Table A3 and Table A4).

### 2.2. Influence of MC-LR Inhalation on Gut Microbial Community

**Figure 2:**
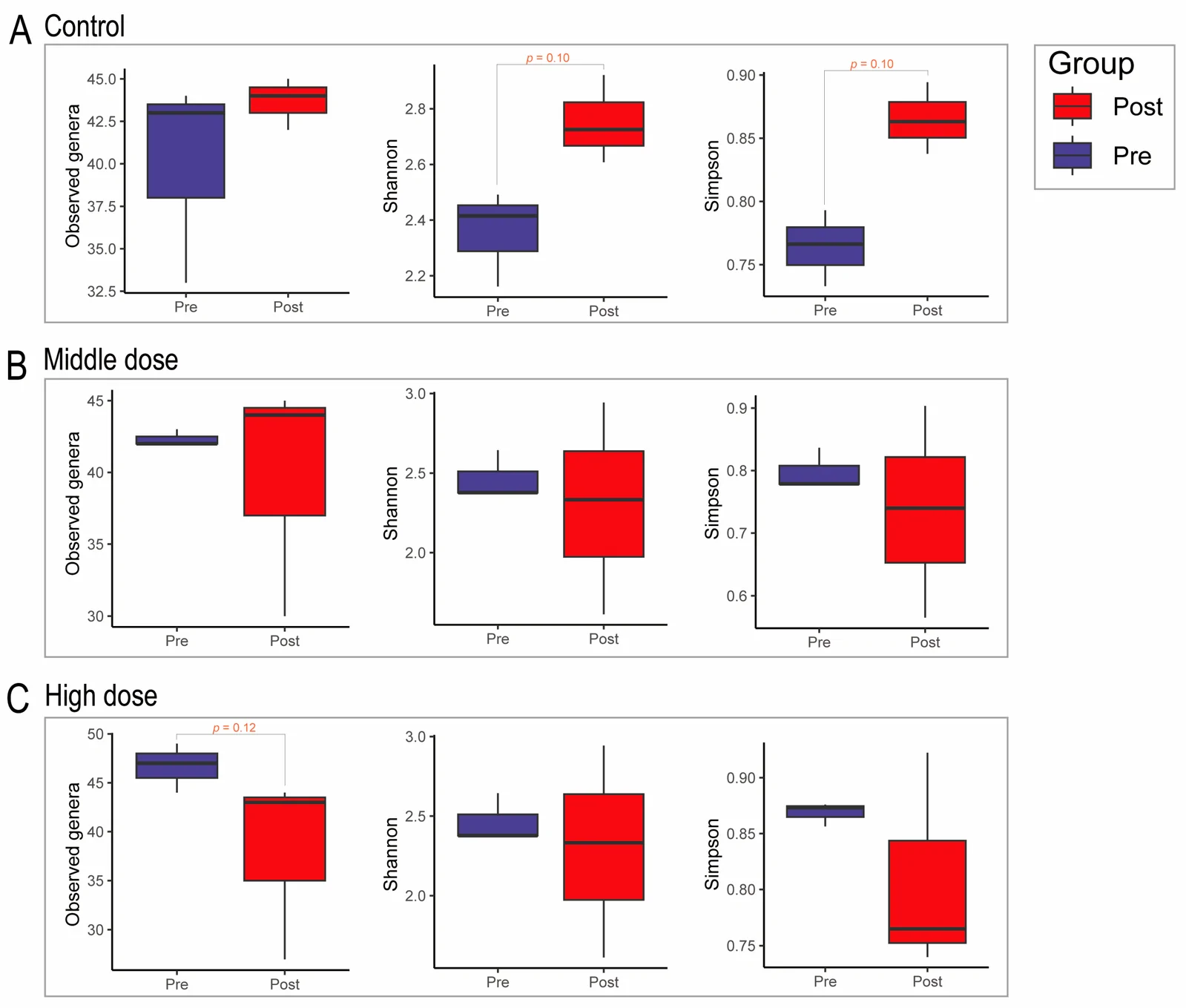
Alpha diversity of mouse gut microbiome (*n* = 5 per group). The change in richness, Shannon, and Simpson diversity before and after microcystin (MC) inhalation is presented, grouped by control (**A**), medium dose (**B**), and high dose (**C**).

To examine gut microbial response to MC inhalation, we measured alpha and beta diversity of gut microbiota before and after the inhalation. First, we calculated three alpha diversity indices (observed genera, Shannon, and Simpson) of mouse gut microbiome at the genus level. In the control group, Shannon and Simpson diversity significantly increased during the experiment duration (72 h) (*p* = 0.10) (Figure 2A). In the high-dose group, a decrease in richness was observed (*p* = 0.12) (Figure 2C). On the other hand, the medium-dose group did not show significant change in alpha diversity after inhalation (Figure 2B).

**Figure A1:**
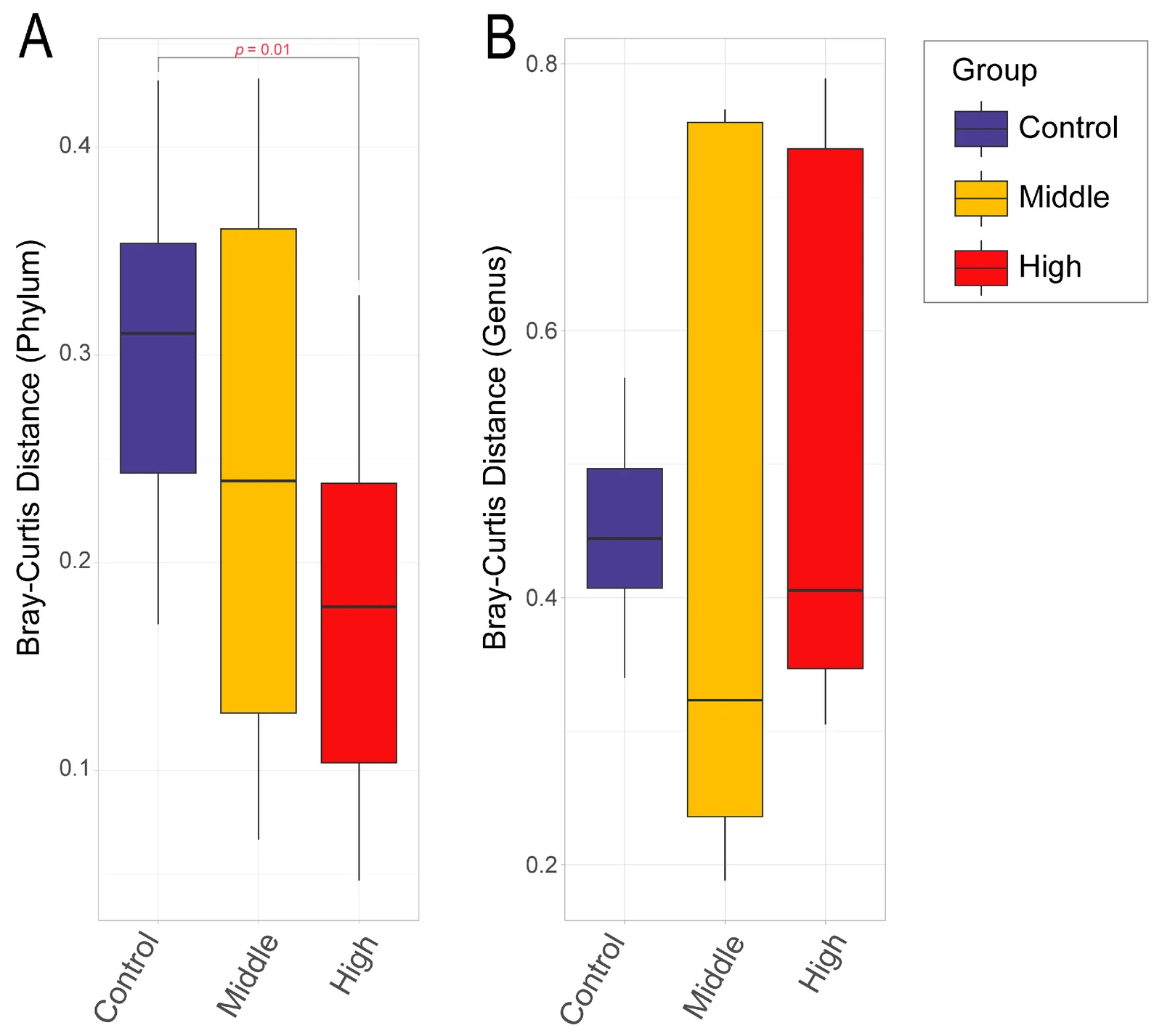
Changes in gut microbial composition following MC inhalation, measured by Bray–Curtis distance between pre- and post-exposure samples. (**A**) Phylum level distance. (**B**) Genus level distance. Boxplots represent the distances measured in control, middle, and high dose groups.

Next, we examined how MC inhalation changed microbial composition by different nasal exposure doses. The extent of alteration in microbial composition was measured by beta diversity (Bray–Curtis distance) between the gut microbiota before and after the inhalation, as the higher beta diversity values indicated the more substantial alteration. We observed significant differences in the post-exposure gut microbial response between the control and high-dose groups (*p* = 0.01), showing that the high-MC-dose group changed less than the control group at the phylum level (Figure A1A). We did not observe a consistent trend at the genus level (Figure A1B).

### 2.3. Prediction of Mouse Gut Microbial Pathway

Finally, we conducted gut microbial metabolic pathway prediction analyses to evaluate the potential effects of MC inhalation on the functional profiles of the gut microbiota and to infer possible health implications for the host associated with these functional alterations. When we selected pathways significantly altered after MC exposure, we used the following criteria: (1) the relative abundance of pathways before inhalation was not significantly different by dose groups; (2) the relative abundance of pathways did not significantly change during the experiment duration in the control group; (3) the relative abundance of pathways in either the medium- or the high-dose group was significantly altered after the inhalation.

Based on these criteria, we identified eight gut microbial pathways significantly increased after MC inhalation. Five pathways out of them, namely Heme b Biosynthesis-II (oxygen-independent) (Heme-Biosynthesis-II), peptidoglycan biosynthesis II (staphylococci) (PWY-5265), sulfur oxidation (*Acidianus ambivalens*) (PWY-5304), and peptidoglycan biosynthesis V (β lactam resistance) (PWY-6470), were significantly increased in both the medium- and high-dose groups (*p* < 0.10) (Table 1). On the other hand, the remaining three of them, namely octane oxidation (P221-PWY), 2,3-butanediol biosynthesis (PWY-6396), and glycerol degradation to butanol (PWY-7003), significantly increased in the medium-dose group only (Table 1).

**Figure 3:**
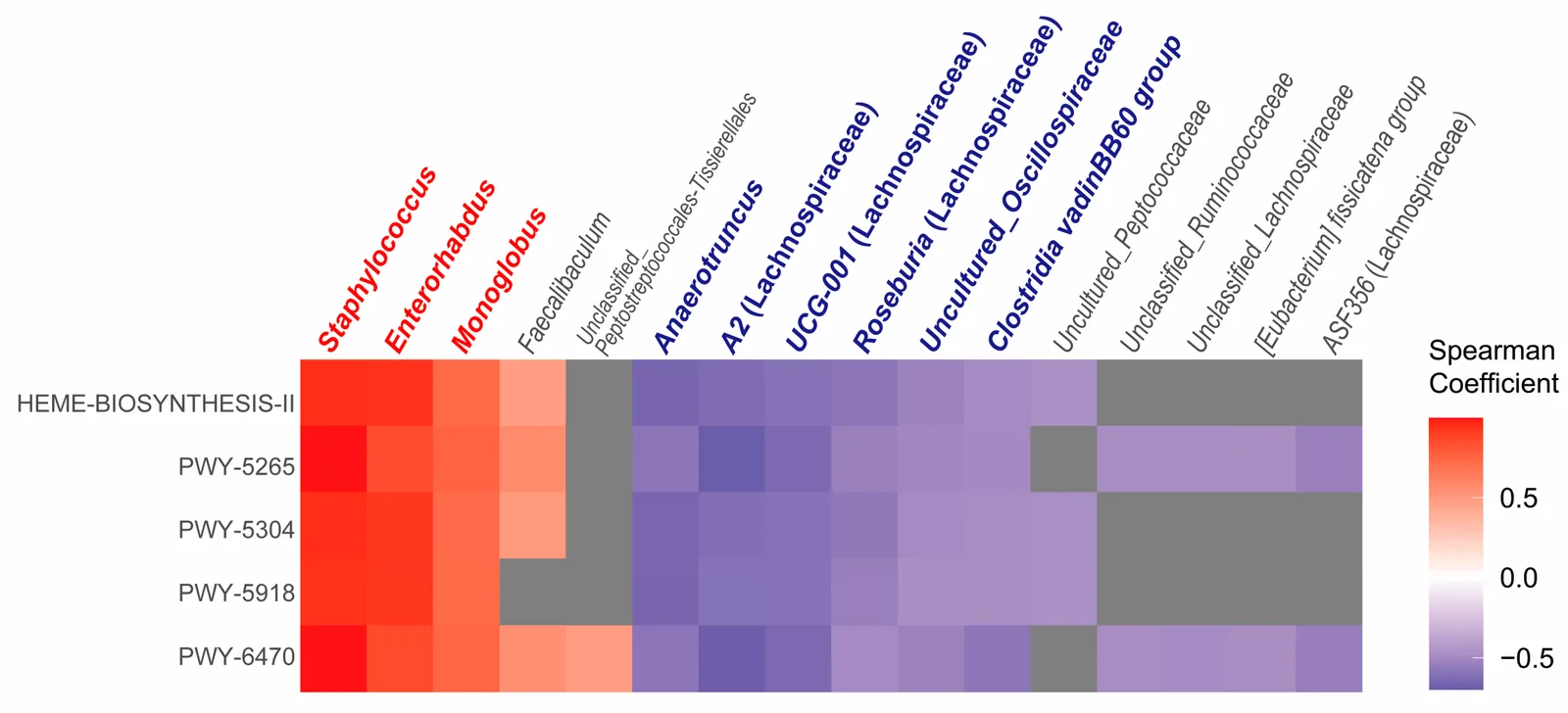
Correlation between the relative abundance of bacterial genera and pathways, predicted by PICRUSt2. Only bacterial genera significantly correlated (Spearman, *p* < 0.05) with the relative abundance of pathways are shown on the heatmap.

Moreover, we identified gut bacterial genera potentially contributing to that functional alteration after MC inhalation. A total of 16 genera were significantly correlated with at least one of the five pathways (Spearman, *p* < 0.05), which significantly increased in the mouse gut after MC inhalation in both the medium- and high-dose groups (Figure 3). Three genera out of them, *Staphylococcus*, *Enterorhabdus*, and *Monoglobus*, were positively correlated with all five pathways (Figure 3). In addition, we identified six genera, namely *Anaerotruncus*, *A2* (Lachnospiraceae), *UCG-001* (Lachnospiraceae), *Roseburia* (Lachnospiraceae), Uncultured Oscillospiraceae, and *Clostridia vadinBB60* group, all of which were significantly negatively correlated (Figure 3).

## 3. Discussion

MC inhalation affects the lower respiratory tract, making BAL immune cell profiles an important readout of pulmonary inflammation. In our study, high-dose MC inhalation increased monocyte numbers in BAL fluid after high-dose exposure, indicating recruitment of inflammatory cells into the alveolar space. Macrophage and total BAL cell counts were not significantly changed, suggesting that MC exposure induces a selective inflammatory response rather than a broad increase in BAL cellularity. These pulmonary immune changes provide context for the broader host responses associated with MC inhalation, including alterations in gut microbial pathways.

The result of our gut microbial pathway prediction implied that gut dysbiosis triggered by MC inhalation may exert adverse effects on host health. For example, in our study, an increase in the pathways related to heme b biosynthesis-II (Heme-Biosynthesis-II and PWY-5918) in the gut was observed after the inhalation. Heme has been reported to be essential for bacterial pathogenesis [41,42] and has been shown to result in gut dysbiosis by oral administration [43]. Additionally, our prediction showed that MC inhalation was related to increased relative abundance of peptidoglycan synthesis pathways (PWY-5265 and PWY-6470), which were associated with bacterial resistance against antibiotics such as vancomycin or beta-lactams [44,45]. Notably, Saha et al. [46] also reported increased relative abundance of antibiotic resistance genes after MC exposure in their mouse study. Given that in the study the authors assumed the exposure route to be ingestion (oral gavage route), the agreement between the study and ours emphasized that inhalation can also be a significant route delivering an effect of MC exposure toward the gut resistome. Additionally, pathways related to sulfur oxidation (PWY-5304) significantly increased in both the medium- and high-dose groups. According to Leleiwi et al. [47], inflammatory conditions in the gut promote microbial sulfur metabolism, during which gut bacteria oxidize sulfur compounds to molecules such as tetrathionate and thiosulfate, which can subsequently create a favorable environment for enteric pathogens.

Finally, we observed an increased relative abundance of several microbial carbon metabolic pathways, including octane oxidation (P221-PWY), 2,3-butanediol biosynthesis (PWY-6396), and glycerol degradation to butanol (PWY-7003). Li et al. [48] demonstrated, using a C57BL/6J mouse model, that high-alcohol-producing *Klebsiella pneumoniae* contributes to the development of fatty liver disease through 2,3-butanediol biosynthesis. In addition, Baumann-Dudenhoeffer et al. [49] reported that increased activity of the glycerol degradation to butanol pathway is a characteristic feature of gut microbial dysbiosis and has been associated with inflammatory disorders, allergies, and fatty liver. Collectively, these findings suggest that the enrichment of carbon metabolic pathways observed following MC inhalation may reflect microbiome functional changes that are linked to metabolic dysfunction and inflammatory disease progression.

Another notable finding was that the responses of these carbon metabolic pathways to MC inhalation exhibited a non-monotonic dose-response pattern. Specifically, the pathways were significantly enriched in the medium-dose group, whereas similar changes were not consistently observed in the high-dose group. Such non-monotonic dose-response relationships are increasingly recognized as common phenomena in toxicology, particularly following exposure to environmental toxicants and other xenobiotics [50,51,52]. Rather than showing a simple linear relationship between dose and biological effect, these responses often exhibit greater effects at intermediate doses than at higher doses. Furthermore, Kamrad et al. [53] demonstrated that gut microbial communities can also display non-monotonic functional responses following xenobiotic exposure. Our findings indicate that microbial carbon metabolic pathways also exhibited non-monotonic responses following MC inhalation.

Such alterations in gut microbial pathways have also been reported in previous studies of inflammatory bowel disease (IBD). For instance, Yu et al. [54] reported increased relative abundance of predicted PWY-5265 (peptidoglycan biosynthesis II) and PWY-6470 (peptidoglycan biosynthesis V) in the gut microbiota of patients with inflammatory bowel disease and Clostridium difficile infection. Interestingly, several of the pathways in our study have also been predicted to increase in hosts with impaired liver function. For example, Heme-Biosynthesis-II and PWY-5918 (heme b biosynthesis from glutamate) were predicted to be enriched in the gut microbiota of patients with nonalcoholic fatty liver disease [55], and these functions in host oral microbiota were reported to be positively correlated with low-density lipoprotein levels [56]. In addition, PWY-5918 (heme b biosynthesis from glutamate) was also predicted to be enriched in the gut microbiota of patients with hepatitis C virus (HCV)-associated cirrhosis [57]. Taken together, these findings suggest that the alterations in gut microbial pathways following MC inhalation may reflect inflammatory responses or tissue damage in distal organs.

Moreover, the gut microbial pathways associated with MC inhalation in this study were significantly correlated with the relative abundance of several gut microbial genera. For instance, the pathways were significantly positively correlated with the relative abundance of *Staphylococcus*, *Enterorhabdus*, and *Monoglobus*, which were related to impaired health status of the host. For example, relative abundance of *Enterorhabdus* in mouse gut was related to autoimmunity and inflammatory bowel diseases [58], and *Staphylococcus* has been reported to be a major intestinal pathogen [59]. On the other hand, bacterial genera, significantly negatively correlated with the pathways, were frequently mentioned to play a commensal or beneficial role in the host gut. For example, Lachnospiraceae was reported to play a role in suppressing colitis [60]; *Clostridia vandinBB60 group* was shown to contribute to the amelioration of colitis by inducing T helper 2 and T regulatory cells in dextran sulfate sodium (DSS)–induced colitis model [61]; a genus in Oscillospiraceae has been reported to be a representative bacteria in the gut of a healthy mouse [62].

Acute perturbations of the gut microbiome are generally considered to be reversible because of the inherent resilience of the gut microbial community, particularly following transient disturbances such as dietary changes [63], diarrhea, or antibiotic treatments [64]. However, increasing evidence suggests that even transient gut dysbiosis can have lasting consequences for host health. First, acute perturbations may leave persistent signatures in the gut microbiome even after apparent recovery. For example, Berry et al. [65] demonstrated in a dextran sodium sulfate (DSS)-induced colitis mouse model that the gut microbiota remained significantly different from that of healthy control mice even after recovery from colitis. Notably, post-recovery microbial composition distinguished mice according to their history of intestinal inflammation more sensitively than conventional pathological markers. Similarly, Schwab et al. [66] reported that the gut microbiota did not fully recover after resolution of DSS-induced colitis. In addition to persistent alterations in microbial composition, recovery of microbial functional potential was also impaired, indicating that previous inflammatory episodes can have long-lasting effects on both the structure and functional capacity of the gut microbiome.

Second, even when the gut microbiota largely recovers, transient dysbiosis may still produce persistent physiological consequences. For instance, Ba et al. [67] gut dysbiosis induced by low-dose cadmium exposure during early life resulted in lasting effects on host health into adulthood. In their C57BL/6J mouse model, mice exposed to cadmium during early life (8 weeks) exhibited increased adiposity in adulthood. Importantly, fecal microbiota transplantation experiments demonstrated that this phenotype was primarily attributable to cadmium-induced gut dysbiosis rather than the direct toxic effects of cadmium. Consistent with these findings, several studies using antibiotic treatment as a transient perturbation have shown that early-life microbial disturbances can have persistent biological consequences long after antibiotic exposure has ceased. These include increased susceptibility to gastrointestinal pathogen infections and subsequent colitis [68], impaired nervous system development [69], and an increased risk of chemically induced colitis [70].

Although the present study did not evaluate the persistence of gut microbiome alterations following MC inhalation, these previous findings suggest that even transient MC-induced dysbiosis may have lasting biological consequences. Longitudinal studies are therefore warranted to determine the duration of microbiome alterations following MC inhalation and their potential implications for long-term host health.

To the best of our knowledge, this study is the first to investigate the effect of MC inhalation on the gut microbiome. Although this exploratory study was limited in elucidating the precise mechanisms by which inhaled MC alters gut microbiota, previous animal studies examining inhaled toxins provide a plausible mechanistic basis for interpretation. For example, Jung et al. [17] showed the potency of MC inhalation in affecting distal organs beyond the respiratory tract crossing over the respiratory epithelium. Furthermore, an animal study by Kim et al. that investigated the effect of cigarette smoking on mouse guts showed increased inflammatory signatures from respiratory tracts and blood from the exposure groups, followed by increased inflammation gene expression from the gut [71]. The study demonstrated that systemic propagation of inflammatory responses by toxic inhalation eventually resulted in inflammation in the gastrointestinal tract. Similarly, Ran et al. [72] showed that PM2.5 inhalation resulted in lung damage along with systemic inflammation and lung and gut dysbiosis.

Taken together, these findings suggest that MC inhalation may influence the gut microbiota through at least two potential pathways. First, inhaled MC may cross the respiratory epithelium and subsequently induce inflammation in distal organs, including the gut, thereby altering the microenvironment. Such changes may contribute to gut dysbiosis through mechanisms similar to those observed following oral MC exposure [46]. Second, even if MC does not translocate beyond the respiratory tract, the local inflammatory responses triggered in the lung may propagate systemically through the lung–gut axis, ultimately contributing to gut dysbiosis. To elucidate the precise mechanisms underlying these effects, further studies are needed to evaluate immune responses across multiple organs and determine the tissue distribution of MC following respiratory exposure. In addition, future studies should evaluate the effects of chronic inhalation at environmentally relevant MC exposure levels to better assess potential health risks.

Our exploratory study suggests that MC inhalation and the subsequent respiratory inflammation responses may contribute to gut microbiome dysbiosis. Given the critical role of the gut microbiome in maintaining long-term host health, our findings highlight the need for follow-up studies investigating the chronic effects of MC inhalation on gut microbial communities and systemic health outcomes. Furthermore, future studies should evaluate how preexisting respiratory conditions, such as asthma and chronic obstructive pulmonary disease, influence the gut microbiome response to MC respiratory exposure.

## 4. Materials and Methods

### 4.1. Experimental Design

All experiments involving mice were conducted with protocols approved by the Institutional Animal Care and Use Committee of The Ohio State University. Fifteen C57BL/6J female mice (8 weeks of age) were purchased from Jackson Laboratories (Bar Harbor, ME, USA) and randomly separated into three groups (five mice per cage): control (no MC); medium dose (25 μg/kg body weight); and high dose (50 μg/kg body weight). Mice were maintained on a 12/12 h light/dark cycle with controlled temperature and humidity and ad libitum access to a standard diet and water. The mice were allowed to acclimate to the animal facility for 1 week prior to the experiment.

For MC, we used microcystin–leucine–arginine (MC-LR), the most frequently reported variant and most toxic among the congeners [73]. It was diluted following provider’s instructions (Cayman Chemical, Ann Arbor, MI, USA) for the subsequent analysis. The doses we used for this study were designed to represent sublethal acute inhalation exposure based on previously published studies. A review of the literature indicated that an intratracheal instillation of 50 μg/kg body weight of MC-LR was associated with the upper range of acute daily respiratory exposure while remaining below the lethal threshold [24]. In addition, cumulative doses used in longer-term exposure studies were taken into consideration. For example, Benson et al. [24] administered a cumulative dose of 217 μg/kg body weight for 7 consecutive days, whereas Oliveira et al. administered a total of 201 μg/kg body weight for 30 consecutive days [25]. Based on these, we selected a cumulative dose of 150 μg/kg body weight over three consecutive days (50 μg/kg body weight/day) as the high dose to reflect sublethal acute exposure. The medium-dose group received half of the dose (25 μg/kg body weight/day; cumulative dose: 75 μg/kg body weight).

All mice were exposed to MC-LR via intranasal injection once a day for 3 days. Mice were lightly anesthetized with isoflurane prior to intranasal instillation. A sublethal dose of MC-LR was administered intranasally in a total volume of 30 μL once daily for three consecutive days. The dosing solution was prepared in sterile 0.9% saline and delivered dropwise to the external nares while the mice were maintained in a supine position, allowing spontaneous inhalation during normal respiration. Following administration, mice were monitored until they had fully recovered from anesthesia. Control mice received an equal volume (30 μL) of sterile 0.9% saline using the identical intranasal instillation procedure.

**Figure 4:**
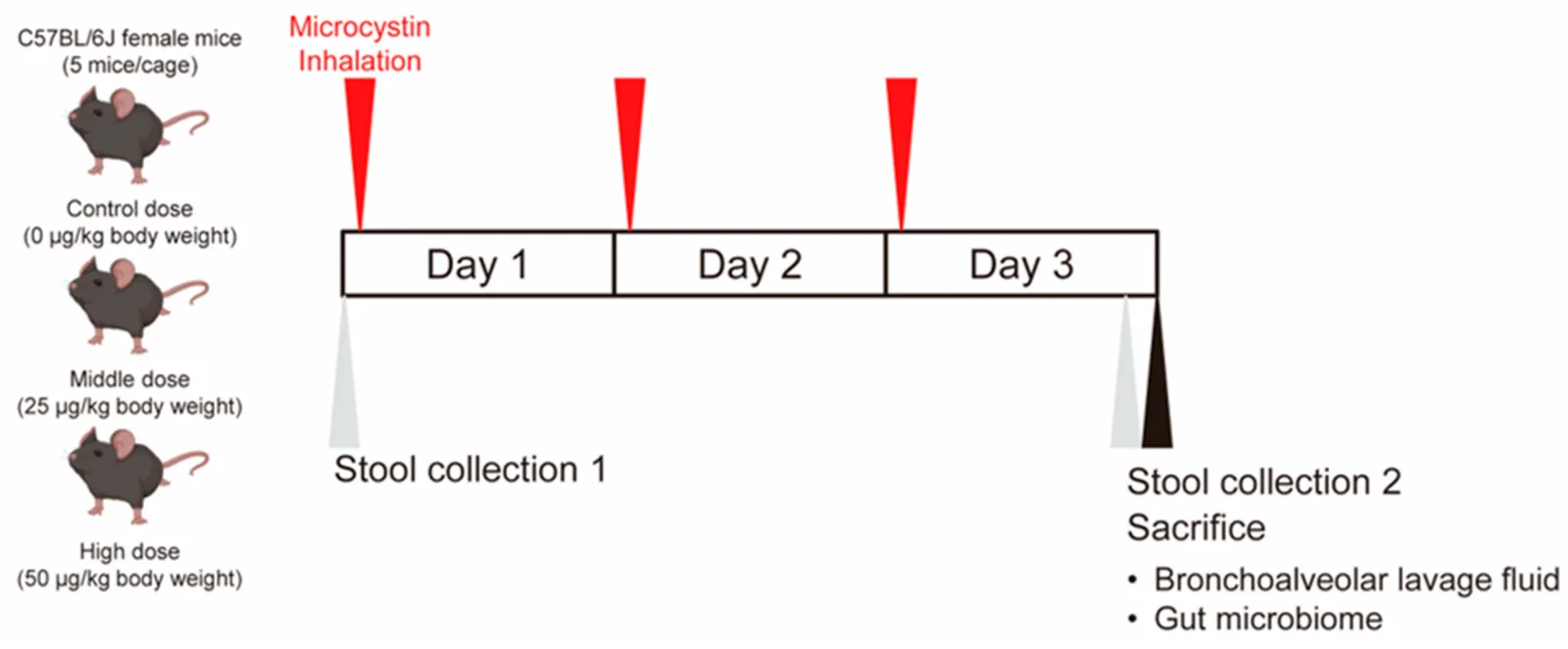
Experimental design.

Fecal samples were collected daily from the cages. Twenty-four hours following the final exposure, mice were euthanized, and lung samples were taken. The collected samples were stored at −80 °C (Figure 4).

### 4.2. Cell Count of Immunocytes in Alveoli

Bronchoalveolar lavage (BAL) fluid was collected to obtain immune cells in the alveolar space. Total BAL cells were counted, and differential immune cell populations, including neutrophils, alveolar macrophages, and monocytes, were analyzed by flow cytometry. BAL cells were incubated with Fc-blocking anti-mouse CD16/32 antibody to reduce nonspecific antibody binding and then stained with fluorochrome-conjugated antibodies against CD45, Ly6G, Siglec F, CD11c, CD3, and CD11b. Samples were acquired using a BD LSRFortessa™ Cell Analyzer (BD Biosciences, Milford, MA, USA). Gating strategies were established based on unstained controls and isotype-matched control antibodies, as previously described (PMID: 42113903). Flow cytometry data were analyzed using FlowJo™ v10 software (BD, Ashland, OR, USA).

### 4.3. Fecal Microbiota DNA Extraction and High-Throughput Sequencing

After thoroughly mixing mouse feces collected at each dose group before and after the MC exposure, we randomly subsampled them in triplicate. Then, DNA extraction was conducted using DNeasy PowerSoil Pro kits (Qiagen, Hilden, Germany) following the manufacturer’s instructions. The quantity and quality of the DNA were measured using a NanoDrop 2000 Spectrophotometer (Thermo Fisher Scientific, Waltham, MA, USA). The prepared DNA samples were sequenced using Illumina MiSeq equipment (Illumina, San Diego, CA, USA), targeting the V4-5 hypervariable region on 16S ribosomal RNA amplified by primer pair: 515F (5′-barcode-spacer-GAGTGCCAGCMGCCGCGGTAA-3′) and 806R (5′-barcode-spacer-ACGGACTACHVGGGTWTCTAAT-3′). Raw data are available at the NCBI Sequence Read Archive (SRA) under the Bioproject PRJNA1451358.

### 4.4. Bioinformatics and Statistical Analyses

Raw data were processed using QIIME2 (version 2022.8.0) [74]. For taxonomy assignment, a classifier trained by the SILVA 138.1 database was used [75]. Amplicon sequence variants (ASVs) classified as ‘Unassigned’, ‘Eukaryota’, ‘Archaea’, ‘Mitochondria’, or ‘Chloroplast’ were removed from the dataset.

To evaluate the effects of MC inhalation on the mouse gut microbiome, we compared microbial alpha diversity and beta diversity among the control, medium-dose, and high-dose groups. We also assessed the associations between MC exposure (control vs. exposed) and exposure dose (medium vs. high) with microbial alpha and beta diversity. For the alpha diversity, richness (number of observed genera), Shannon, and Simpson indexes were used as indicators. All alpha diversity metrics were calculated using R package ‘vegan’ 2.7-3 [76]. Additionally, PICRUSt2 software (version 2.5.2) was used for metagenome function prediction to infer the effects of MC inhalation on gut microbial pathway alterations [77]. To evaluate the potential functional implications of MC-induced changes in the gut microbiome, predicted microbial metabolic pathways were generated using PICRUSt2. PICRUSt2 infers the functional potential of microbial communities by phylogenetically placing amplicon sequence variants (ASVs) onto a reference tree, estimating gene family abundances and 16S rRNA gene copy numbers from the nearest reference bacterial and archaeal genomes, and mapping the predicted gene families to metabolic pathways [77].

The Kruskal–Wallis test (global test) and Dunn test with Benjamini–Hochberg correction (post-hoc) using R’s basic functions were employed to measure the statistical strength of the differences in cell counts of immunocytes in BAL fluids between MC dose groups. Spearman correlation coefficient between predicted microbial pathways and bacterial abundances was calculated using the R package ‘hmisc’ 5.2-6 [78]. Figures were generated using the R package ‘ggplot2’ 4.0.3 [79].

## Figures and Tables

**Table 1:** Pathways indicating significant changes before and after the inhalation. The ones significant in both the medium- and high-dose groups are highlighted in bold.

Pathway	Description	*p*-Value (Pre vs. Post)	
		Medium Group	High Group
**Heme-Biosynthesis-II**	**Heme b biosynthesis II (oxygen-independent)**	**0.06**	**0.08**
**PWY-5265**	**Peptidoglycan biosynthesis II (staphylococci)**	**0.06**	**0.06**
**PWY-5304**	**Sulfur oxidation (*Acidianus ambivalens*)**	**0.06**	**0.08**
**PWY-5918**	**Heme b biosynthesis from glutamate**	**0.06**	**0.08**
**PWY-6470**	**Peptidoglycan biosynthesis V (β lactam resistance)**	**0.06**	**0.06**
P221-PWY	Octane oxidation	0.06	0.51
PWY-6396	2,3-butanediol biosynthesis	0.06	0.20
PWY-7003	Glycerol degradation to butanol	0.07	0.64

## Data Availability

The data presented in this study are openly available in the NCBI Sequence Read Archive (SRA) under the bioproject PRJNA1451358.

## Abbreviations

The following abbreviations are used in this manuscript:

ASV	Amplicon sequence variant
BAL	Bronchoalveolar lavage
MC	Microcystin
MC-LR	Microcystin–leucine–arginine

## Appendix A

**Table A1:** Cell count of neutrophils in BAL fluids by MC doses. The descriptive statistics (mean, sample number, standard deviation, median, Q1 (25th percentile), and Q3 (75th percentile)) of neutrophil counts in BAL fluids were summarized by dose groups.

Dose	*n*	Mean	SD	Median	Q1	Q3
Control	5	2.64 × 10^3^	1.25 × 10^3^	2.70 × 10^3^	1.80 × 10^3^	3.00 × 10^3^
Medium	5	1.33 × 10^4^	1.12 × 10^4^	1.55 × 10^4^	2.56 × 10^3^	1.71 × 10^4^
High	5	1.89 × 10^4^	1.63 × 10^4^	1.78 × 10^4^	4.84 × 10^3^	3.40 × 10^4^

**Table A2:** Cell count of monocytes in BAL fluids by MC doses. The descriptive statistics (mean, sample number, standard deviation, median, Q1 (25th percentile), and Q3 (75th percentile)) of neutrophil counts in BAL fluids were summarized by dose groups.

Dose	*n*	Mean	SD	Median	Q1	Q3
Control	5	2.43 × 10^2^	2.56 × 10^2^	2.07 × 10^2^	1.80 × 10^3^	3.00 × 10^3^
Medium	5	6.10 × 10^3^	7.20 × 10^3^	5.90 × 10^3^	1.50 × 10^2^	6.26 × 10^3^
High	5	1.30 × 10^4^	1.30 × 10^4^	1.21 × 10^4^	4.79 × 10^2^	2.22 × 10^4^

**Table A3:** Count of total cells in BAL fluids by MC doses. The descriptive statistics (mean, sample number, standard deviation, median, Q1 (25th percentile), and Q3 (75th percentile)) of neutrophil counts in BAL fluids were summarized by dose groups.

Dose	*n*	Mean	SD	Median	Q1	Q3
Control	5	1.49 × 10^5^	5.59 × 10^4^	1.36 × 10^5^	1.20 × 10^5^	1.69 × 10^5^
Medium	5	1.37 × 10^5^	4.48 × 10^4^	1.52 × 10^5^	9.24 × 10^4^	1.69 × 10^5^
High	5	1.85 × 10^5^	5.65 × 10^4^	1.90 × 10^5^	1.58 × 10^5^	2.07 × 10^5^

**Table A4:** Cell count of alveolar macrophages in BAL fluids by MC doses. The descriptive statistics (mean, sample number, standard deviation, median, Q1 (25th percentile), and Q3 (75th percentile)) of neutrophil counts in BAL fluids were summarized by dose groups.

Dose	*n*	Mean	SD	Median	Q1	Q3
Control	5	8.08 × 10^4^	1.09 × 10^4^	8.21 × 10^4^	8.09 × 10^4^	8.67 × 10^4^
Medium	5	8.23 × 10^4^	1.96 × 10^4^	8.80 × 10^4^	6.31 × 10^4^	9.22 × 10^4^
High	5	8.94 × 10^4^	2.19 × 10^4^	7.98 × 10^4^	7.38 × 10^4^	1.12 × 10^5^
